# Forest Fire Detection via Feature Entropy Guided Neural Network

**DOI:** 10.3390/e24010128

**Published:** 2022-01-15

**Authors:** Zhenwei Guan, Feng Min, Wei He, Wenhua Fang, Tao Lu

**Affiliations:** 1 Hubei Key Laboratory of Intelligent Robot, School of Computer Science and Engineering, Wuhan Institute of Technology, Wuhan 430073, China; 22007010007@stu.wit.edu.cn (Z.G.); fangwh@whu.edu.cn (W.F.); lut@wit.edu.cn (T.L.); 2 College of Physics and Electronic Information Engineering, Minjiang University, Fuzhou 350108, China

**Keywords:** forest fire detection, feature entropy, convolutional neural network

## Abstract

Forest fire detection from videos or images is vital to forest firefighting. Most deep learning based approaches rely on converging image loss, which ignores the content from different fire scenes. In fact, complex content of images always has higher entropy. From this perspective, we propose a novel feature entropy guided neural network for forest fire detection, which is used to balance the content complexity of different training samples. Specifically, a larger weight is given to the feature of the sample with a high entropy source when calculating the classification loss. In addition, we also propose a color attention neural network, which mainly consists of several repeated multiple-blocks of color-attention modules (MCM). Each MCM module can extract the color feature information of fire adequately. The experimental results show that the performance of our proposed method outperforms the state-of-the-art methods.

## 1. Introduction

At present, a large number of forest fire accidents occur every year and cause serious economic losses. For example, the Australian fire event brought about serious disasters, not only causing a lot of economic losses, but also causing a lot of biological casualties. In order to make an alarm for fire accidents, most of the current practices are based on fire alarm systems. However, most of these methods are based on sensors. Unfortunately, sensor-based methods may not be accurate enough to determine whether there is fire in the real world. For example, smoke-based sensors may have false triggers [1]. In addition, these sensors are generally widely deployed in indoor scenes and are not suitable for large-scale outdoor environments, such as forests. Moreover, some researchers have used vision-based methods to solve the problem of indoor fire early warning, and have made excellent progress. However, due to the distance and scale reasons, the performance of these methods directly used in outdoor forest scenes are seriously degraded. Therefore, vision-based methods for early warning systems of forest fires is still a challenging task.

The vision-based approach directly obtains images from the camera to immediately judge whether there is a fire. As a result, these vision-based methods can provide a warning at the early stage of fire, which is of great significance for the early warning of the forest fire. Therefore, some methods have been proposed based on computer vision. These methods can be divided into two methods according to the expression of features, including traditional manual feature extraction methods for forest fire detection and forest fire detection methods based on deep learning (DL) [2].

Traditionally, the researchers attempted to design methods of manually extracting features for fire detection. These methods use manual features in feature expression. In [3], Liu et al. proposed a new flame detection algorithm that is based on a saliency detection technique and on the uniform local binary pattern (ULBP). In order to utilize the color information of the flame, they think the probability density function (PDF) of the flame pixel color can be obtained using Parzen window nonparametric estimation. Experimental results show the effectiveness of the method. In another work [4], a method based on multifeature fusion of the flame was proposed by Gong et al. At the preprocessing stage, it involves motion detection and color detection of the flame. They proposed a algorithm of flame centroid stabilization based on the spatiotemporal relation because of a certain similarity in the sequence of the image. Furthermore, in [5], Li et al. proposed an approach which considers a set of parametric representations called Gradient Features (GF) in order to learn the features of flame color changes in the image. In contrast to the traditional color features of the flame, GF represents the color changes in RGB channels. They also used a support vector machine to generate a set of candidate regions and adopted the decision tree model to judge flame regions based on GF. According to their experiment, their approach can distinguish between yellow color light and sunrise scenes. Some researchers also adopted approaches based on the motion and color properties. In [6], a novel method based on the chromatic and dynamic properties of the fire was proposed by Thou-Ho et al. In [7], Rinsurongkawong et al. proposed an approach for flame detection based on the dynamic properties. However, the approach has an apparent defect because it failed with images with similar features with fire. In addition, these methods need to be cautious while extracting features in order to obtain accurate features. However, in the face of such a large amount of data that need to be processed, manual feature extraction will become impractical because the expression ability of manually extracted features is insufficient. Furthermore, the handcrafted approaches show poor performance, and it is unreliable because of low accuracy.

Currently, methods based on deep learning (DL) are in the mainstream. In [8], Zhang et al. proposed a DL method for forest fire detection. They trained both a full image and fine grained patch fire classifier in joined deep convolutional neural networks. Their method first judges whether there is a fire through a full image, and if it is determined that there is a fire, they then conduct a fine-grained and accurate location procedure. In [9], Sharma et al. proposed a deep neural network for fire detection. They fine-tuned the Vgg16 [10] and ResNet50 [11] networks and achieved high accuracy. In addition, various variants of CNNs such as AlexNet [12], GoogleNet [13], and SqueezeNet [14] were proposed for fire detection by Khan Muhammad et al. However, these methods do not take into account the complexity difference between training samples, and they treat training samples with different entropy and complexity equally for classification loss, which limits the performance of their training models. In addition, Jadon et al. proposed a lightweight neural network architecture in [1]. With limited parameters, the performance may be poor in real complex fire scenes. In a word, these methods fail to consider the content complexity differences of different fire scenes, but treat the training samples equally, which reduces the performance of the network model.

In this paper, considering the content complexity difference of training samples has a great impact on the performance of the network model, we propose a novel feature entropy guided neural network for forest fire detection. In order to balance the difference in entropy and complexity of training samples, a cross entropy loss function guided by feature entropy is proposed by us. The larger the sample entropy, the more complex the sample is, and the greater the weight of the corresponding classification loss during training. In addition, we also propose a network based on color attention, which includes several repeated multiple-blocks of color-attention modules (MCM) to fully extract the feature information of the forest fire. We named it “FireColorNet”. Based on FireColorNet, a forest fire detection algorithm is proposed by us. The experimental results prove our point of view, and our method also achieves superior performance. The main contributions of this work are as follows:

1. We propose a cross entropy loss function guided by feature entropy, which is used to balance the difference of content complexity of training samples.

2. A novel color attention network is proposed by us, named FireColorNet. It includes several repeated multiple blocks of color-attention modules (MCM) to fully extract the feature information of the fire, which can be effectively embedded into the forest fire detection algorithm.

3. We propose a forest fire detection algorithm based on the proposed FireColorNet.

## 2. Method

In this part, we first describe the proposed cross entropy loss function based on feature entropy. Secondly, we introduce our color attention neural network FireColorNet. Finally, we introduce the forest fire detection algorithm based on FireColorNet.

### 2.1. Cross Entropy Loss Function Guided by Feature Entropy

The basic function of information entropy is to reflect the uncertainty of things. The general definition of entropy is as follows.

(1) Entropy=−∑P(i)logP(i)

**Figure 1:**
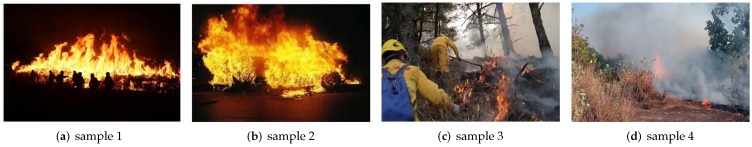
The figure shows the training samples with different content complexity. The entropy value of sample 1 and sample 2 is small, and the image is easier to distinguish as fire; Sample 3 and sample 4 have larger entropy values relatively, and the images are not easy to distinguish as fire.

In Formula (1), P(i) represents the output probability function. Similarly, this can also be used for image information entropy, where P(i) represents the probability of pixel value *i*. This formula describes the content complexity of the image. The more complex the content of the image, the higher the entropy, which means that it is a hard sample to some extent. As a result, it is relatively difficult to determine its category. This idea is of great significance for the classification task. For example, in Figure 1, the information entropy value in sample 1 and sample 2 is smaller because their background content is very simple. On the contrary, for sample 3 and sample 4, their entropy is relatively larger, with relatively complex content. The entropy of the tested samples is shown in Table 1.

Motivated by this view, we propose a cross entropy loss based on feature entropy guidance, which is used to give greater loss weight to difficult samples the category of which is not easy to determine. Specifically, we obtain the last layer feature map of the network. P(i) is the occurrence probability of each value in the last layer of the feature map. The more complex the sample, the greater the feature entropy, the greater the corresponding amount of information, and the greater the corresponding uncertainty. Therefore, greater weight should be given when calculating the loss function for the classification task. Combined with the cross entropy loss function, we can obtain the following Formula (2), which we call the cross entropy loss function guided by feature entropy (*FLoss*).

(2) $$FLoss=-CE_{loss}*\sum P\left(i\right)logP\left(i\right)$$

### 2.2. FireColorNet

**Figure 2:**
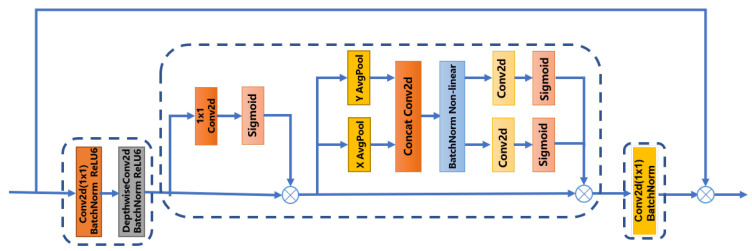
Our “MCM” module. The size of all convolution kernels is 1 × 1 except for depth separable convolution in Figure 2.

We propose a neural network named FireColorNet based on a color-attention mechanism for forest fire detection. The network mainly includes several repeated similar “MCM” modules, and each “MCM” module includes four parts, which is shown in Figure 2. Part one: a 1 × 1 convolution, normalization, activation function and a deep separable convolution. Part two: a color-attention mechanism module. Part three: a 1 × 1 convolution and normalization. Part four: a shortcut.

In the network structure, the depth of separable convolution is to reduce the amount of calculation. At the same time, the color attention mechanism module pays more attention to the color information of the fire. Specifically, the color attention consists of two network modules in series. The former includes a 1 × 1 convolution, a sigmoid activation function and a shortcut, which we named the “*PA*” module. It has proved its superior performance in the paper [15]. Because 1 × 1 convolution can traverse each pixel in the feature map, the color feature information of any pixel can be extracted well. On the surface, it seems simple structure which consists of a 1 × 1 convolution layer and a sigmoid function. Intuitively, the input multiplies the result of the convolution layer and a sigmoid function. The latter includes the coordinate attention mechanism module [16], which we named the “*CA*” module. It can make up for the loss of the spatial location feature information, so as to better determine the location of the fire. Specifically, it can extract the block feature information of the feature maps through the average pooling operation along the horizontal and vertical directions first. Then the feature information after the pooling operation are spliced and merged by a 1 × 1 convolution. In addition, by a 1 × 1 convolution and a sigmoid function, it separates the extracted feature information of the two dimensions. Finally, it performs a shortcut operation. This process can be illustrated by the Formula (3).

(3) $$F_{out}\left(X\right)=F_{CA}\left(F_{PA}\left(X\right)\right)$$

In the equation, *X* represents the input, $X^{N\times C\times W\times H}$. *N* denotes batch-size, and *C* denotes channels. *W* and *H* denote the width and height of the feature maps. The $F_{CA}$ function represents the *CA* module, and $F_{PA}$ represents the *PA* module. $F_{out}$ function denotes the output features. In addition, the 1 × 1 convolution in the third part is to reduce the number of channels, and the shortcut in the fourth part is to incorporate the initial feature information.

The proposed network architecture FireColorNet finally includes a 3 × 3 convolution to extract shallow feature information, named “Conv Block”. Then it consists of seven sub-modules in series, and each sub-module includes several repeated “MCM” modules. In addition, the depth separable convolution in each sub-module is composed of 3 × 3 and 5 × 5 convolution kernels alternately.

### 2.3. Forest Fire Detection Algorithm Based on FireColorNet

**Figure 3:**
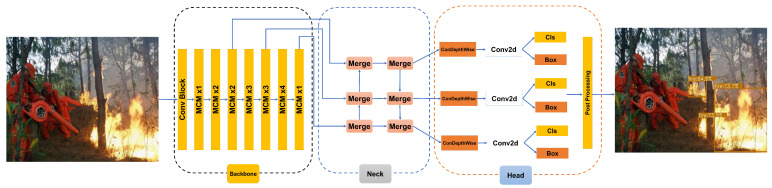
Overview of the forest fire detection algorithm using the proposed FireColorNet as the backbone, which contains three parts, Backbone, Neck and Head. Backbone mainly includes multiple blocks of color attention modules “MCM”, and the Neck is a scale feature fusion “PAN” structure, and the Head is used for prediction. The “Conv Block” represents convolution, normalization, and activation function operations. “× k” represents the number of repetitions of this module. Post processing represents non-maximum suppression operation in the inference phase.

We adopt the proposed FireColorNet as the backbone of the detection algorithm. Moreover, the neck part of our detection algorithm adopts the modified PANet [17] structure. It first performs a semantic information fusion on the extracted features from top to bottom, and then goes through a bottom-up feature fusion to fuse the location and space information. The head part of the detection algorithm adopts NanoDet [18], which is a single-stage anchor-free object detection model that uses the *GFL* [19] loss function to perform classification and border regression. The overall flow chart is shown in Figure 3. *GFL* can be considered as a general term of *QFL* (Quality Focal Loss) and *DFL* (Distribution Focal Loss) loss functions. The loss functions *QFL* loss, *DFL* loss and *GFL* loss are defined as follows.

(4) $$QFL\left(\delta \right)={-|y-\delta |}^{\beta }\left(\left(1-y\right)log\left(1-\delta \right)+ylog\left(\delta \right)\right)$$

(5) $$DFL\left(S_{i},S_{i+1}\right)=-\left(\left(y_{i+1}-y\right)log\left(S_{i}\right)+\left(y-y_{i}\right)log\left(S_{i+1}\right)\right)$$

(6) $$GFL\left(p_{yl},p_{yr}\right)=-{\left|y-\left(y_{l}p_{yl}+y_{r}p_{yr}\right)\right|}^{\beta }\left(\left(y_{r}-y\right)log\left(p_{yl}\right)+\left(y-y_{l}\right)log\left(p_{yr}\right)\right)$$

In the Formula (4), it is one of the extended forms of Focal Loss [20] on the continuous label, and y represents the quality label, while δ denotes the output of sigmoid for classification. Specifically, *y* represents the IOU score of the positive sample. Note that the IOU value is the ratio of the intersection over union between the predicted bounding box and its corresponding ground-truth bounding box. The *DFL* loss takes into account the true distribution usually not too far from the labeling position, and at the same time enables the network to focus more quickly near the labeling position. Furthermore, in the Formula (5), $y_{i}$ and $y_{i+1}$ represent the two interval values after discretizing the continuous integral. Meanwhile, $S_{i}$ and $S_{i+1}$ represent the values corresponding to $y_{i}$ and $y_{i+1}$ after softmax operation. In the Formula (6), it can be understood as assuming that a model estimates the probability of two variables $y_{l}$ and $y_{r}$ as $p_{yl}$ and $p_{yr}$, then finally it uses a linear combination $\hat{y}$ = $y_{l}$$p_{yl}$ + $y_{r}$$p_{yr}$ as prediction, and the corresponding continuous label y for the prediction $\hat{y}$ also meets $y_{l}$≤ y ≤$y_{r}$.

## 3. Dataset Preparation

**Figure 4:**
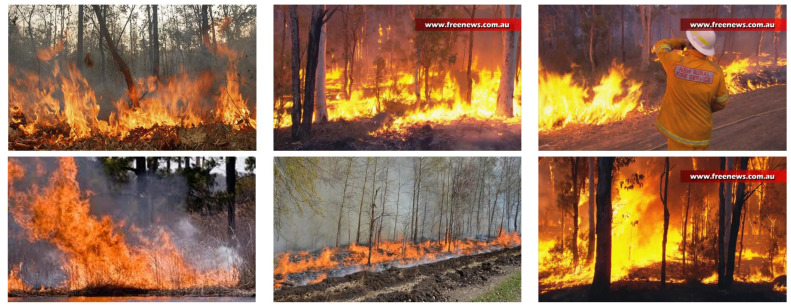
A few sample images from our created dataset.

For the sake of fairness, we used a public fire detection dataset [21] to compare with some other fire detection methods. Specifically, in the public data set, we merge “Neural” and “Smoke” category images into the “NoFire” category, and other images are classified as “Fire” category. Finally, we obtain 2700 training set images and 300 test sets images. In addition, in order to move closer to the forest fire scene, we create a forest fire detection data set based on the images format. We use existing network resources and some video clips to create a forest fire detection data set. Most of the images are in the forest fire scenes in our dataset, and some fire images of other scenes are also included in order to increase the diversity of the dataset. Some dataset samples of our current data set are shown in Figure 4. The data set includes 2200 images, of which there are 1800 images in the training set, 200 images in the validation set and 200 images in the test set. In addition, we also produce a merged data set, which contains our dataset and another fire detection dataset [22]. This merged data set includes training set, validation set and test set, and their numbers are 3735, 500, and 651, respectively. Finally, we chose to use public data sets and merged data sets to compare with some existing fire detection algorithms based on the classification idea. The data set we made is used to compare with some general object detection algorithms based on the detection task.

## 4. Experiments

We used PyTorch framework to implement all experiments. During training, we adopted the SGD with the momentum of 0.9 as our optimizer and the weight decay was 0.0001. The initial learning rate was 0.1. Moreover, we used NVIDIA GPU for training and the batch size was set to 16. In the experiment, in order to be as close to the real scene as possible, we used our created dataset to compare the performance with the general object detection algorithms. At the same time, we also compared with some fire detection algorithms.

### 4.1. Experimental Results

Considering that our network is modified by Efficientnet-b0 [23], we chose the SE module in Efficientnet-b0 as the baseline. Our experimental results are shown in Table 2. From the table, we found that the performance of only using the PA module has exceeded the default SE module. However, the performance of it is still inferior to our color-attention MCM module. It also proves that our proposed MCM module can better be used for feature extraction of fire color. During our experiments, we adopt a serial mode instead of parallel mode or various variants of parallel mode. Detailed experiments are carried out in the ablation studies.

### 4.2. Ablation Studies

**Figure 5:**
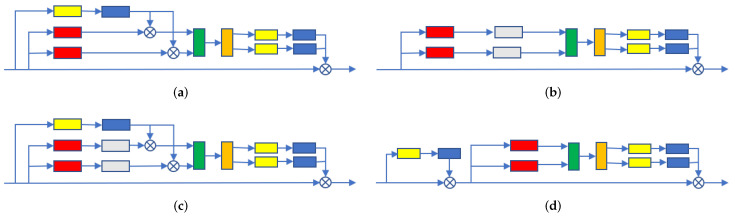
The above figure shows our four different network connection methods. Among them, the yellow box represents 1 × 1 convolution, and the red box represents mean pooling along the horizontal and vertical direction, and the blue box is marked with the sigmoid activation function, and the green represents feature splicing and a 1 × 1 convolution operation, and the orange represents normalization and activation function, and the gray represents our PA module. (**a**) variant1; (**b**) variant2; (**c**) variant3; (**d**) variant4.

In this part, we design four possible connection modes of color attention modules. The four combinations methods can be shown in Figure 5. As can be seen from the figure, the embedding methods of the four color attention modules are very distinctive. In the first and third variants, the initial input module feature information is integrated into the mean pooled feature map after 1 × 1 convolution and sigmoid function processing. However, the second variant is first processed by the average pooling operation along the horizontal and vertical directions, and then processed by the PA module. The fourth method is a series system, which is first processed by the PA module, and then followed up. We think that the performance of different combinations will differ, and the experimental results prove our idea.

In order to study which possible combination method performs best, we executed all our mentioned above method as ablation experiments. All experiments are implemented in our created dataset. The experimental results are shown in Table 3. From the experimental results, we can see that the series mode performs best, so we use this series mode.

### 4.3. Comparison with Other Methods

**Figure 6:**
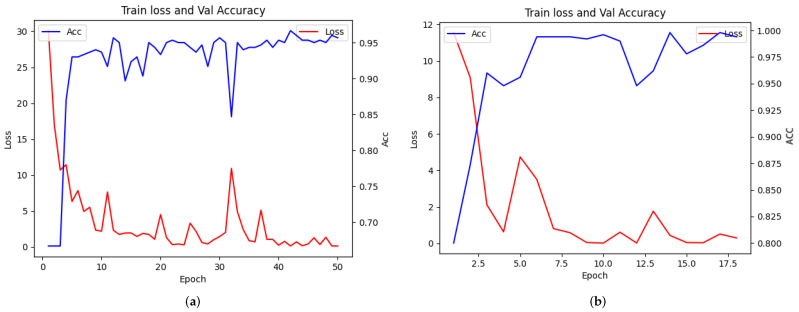
The above figure shows the curves of loss and accuracy with the number of training times in the training phase. The loss function adopts our cross entropy loss based on feature entropy guidance. (**a**) Variation curve of loss and accuracy for the training phase in dataset 1. (**b**) Variation curve of loss and accuracy for the training phase in dataset 2.

In this part, we compare with some fire detection algorithms, and then compare with some general object detection algorithms. Some fire detection algorithms are used to make a comparison, such as FireNet [1], fire detection [9] based on VggNet16 and ResNet50 and fire detection [13] based on GoogleNet. In addition, we use the following criteria to evaluate the performance of the algorithm:

(7) $$Accuracy=\frac{TP+TN}{TP+FN+FP+TN}$$

(8) $$Precision=\frac{TP}{TP+FP}$$

(9) $$Recall=\frac{TP}{TP+FN}$$

where *TP* is the number of true positives, i.e., the number of fire which are classified as fire. *TN* is the number of true negatives, i.e., the number of samples which are correctly classified as the “not fire” category. *FP* represents the number of samples that are incorrectly classified as positive, i.e., the “not fire” category is predicted to be the fire category. Meanwhile, *FN* represents the number of samples that are incorrectly classified as negative, i.e., the number of samples which are predicted to be in the “not fire” category. In addition, we use *Accuracy* as our final evaluation criterion to comprehensively evaluate the performance of the neural network. At the end of each epoch, we perform a verification on the valid set. If the current accuracy on the validation set is higher than the previous accuracy, the current model weight is retained. Our method adopts proposed cross entropy loss function guided by feature entropy. The loss and accuracy curves with epochs in this process are shown in Figure 6.

General detection algorithms include Yolov5(s) [25], Faster-RCNN [26], Grid R-CNN [27], ATSS [28]. In addition, we also compare our method with the Yolov3 [29] algorithm which performs best in this paper [30].

All experimental results can be shown in Table 4 and Table 5. It should be noted that the experimental results in Table 4 are based on the COCO evaluation criteria for object detection. From the experimental results, we can obtain the following conclusions. Overall, our method achieves the highest accuracy on both data sets. On the public data set of dataset 1, the accuracy of our method exceeds other methods. In addition, the accuracy of our cross entropy loss function based on feature entropy guidance (FLoss) is higher than our default cross entropy loss function, which also proves the validity of feature entropy (FLoss). At the same time, it illustrates well that, the larger the entropy value, the more complex the sample, and they are more likely to be complex and difficult samples at the feature level, so we should give more weight to the classification loss in the training phase to improve the performance of the network. In addition, our method (Entropy) on the merged dataset2 reaches the maximum in precision rate and accuracy rate. Compared to some general object detection algorithms, our method surpasses Yolov5(s), ATSS, Faster-RCN, Grid R-CNN and Yolov3, which also indicates that general object detection algorithms may perform poorly if directly used for forest fire detection.

### 4.4. Visualize Prediction Results

**Figure 7:**
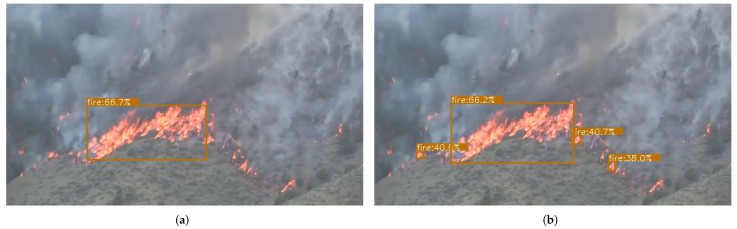
The figure on the left is the visualized result of the default SE module of Eficientnet-b0, and on the right is our MCM module. We can see that our method can detect small and less obvious flames, but the default SE module fails to detect them. We use the same parameters in the detection phase. (**a**) Default SE module; (**b**) our MCM module.

**Figure 8:**
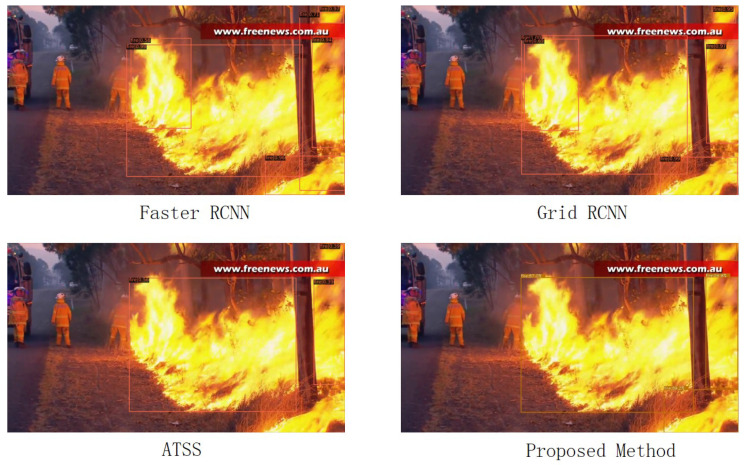
Compared to our method, faster rcnn and grid rcnn have redundant prediction, and atss has a missing prediction at the flame in the lower right corner of the figure.

In this part, we show some prediction results between our method and other detection algorithms. By contrast, our method can detect higher-grained fire and each group of separated fire can be detected independently. As is shown in Figure 7, our method can predict forest fire more accurately compared with the default SE module. Therefore, it can provide more fire information, which is of great significance to forest fire prevention and early warning. Comparison of our algorithm and some other algorithms can be shown in Figure 8. For instance, ATSS preforms poorly and it has a missing prediction for fire in the lower right corner of the figure. In contrast, our method achieves a good balance for prediction.

## 5. Conclusions

Forest fire detection and early warning is an important topic. Around this topic, we propose a novel feature entropy guided neural network for forest fire detection. Feature entropy is used to balance the difference of forest fire samples with different content complexity. The larger the feature entropy, the more complex the image is, and greater weight should be given in the calculation of classification loss. In addition, a novel network named FireColorNet based on color attention is also proposed by us, which can fully extract the color feature information of forest fire. Finally, we also propose a forest fire detection algorithm based on FireColorNet. Given the importance of forest fire detection, we will consider more reliable algorithms for forest fire detection in the following research.

## Figures and Tables

**Table 1:** Information entropy table of test samples.

Samples	Sample 1	Sample 2	Sample 3	Sample 4
**image information entropy**	15.0934	19.5073	22.9191	22.3601

**Table 2:** The experimental results of our MCM module, PA module and the default SE module. All results are based on the COCO [24] evaluation criteria for object detection. The best figure of each metric are highlighted in bold.

Settings	AP		AP50		AP75
**SE/Baseline**	0.422		0.781		0.409
**PA**	0.446		0.821		0.415
**MCM(Ours)**	**0.465**		**0.828**		**0.463**

**Table 3:** Four possible connection modes. All algorithms are implemented based on our 2200 images dataset. All results are based on the COCO evaluation criteria for object detection. The best figure of each metric are highlighted in bold.

Settings	AP	AP50	AP75
**Variants1/Ours**	0.426	0.791	0.404
**Variants2/Ours**	0.434	0.796	0.426
**Variants3/Ours**	0.426	0.767	0.426
**Variants4/Ours**	**0.465**	**0.828**	**0.463**

**Table 4:** Comparison with some other general detection algorithms. All algorithms are implemented on our 2200 images dataset. The best figure of each metric are highlighted in bold.

Algorithm	AP	AP50	AP75
**Yolov3**	0.407	0.767	0.413
**Yolov5(s)**	0.383	0.727	0.357
**Faster-RCNN**	0.433	0.784	0.432
**Grid R-CNN**	0.434	0.781	0.420
**ATSS**	0.432	0.800	0.398
**Proposed Method**	**0.465**	**0.828**	**0.463**

**Table 5:** This table shows the experimental results of our and other methods. Among them, dataset1 represents the public data set. Dataset2 represents our merged dataset. All methods are implemented on the two datasets. We use some evaluation metrics, such as precision, recall, accuracy. However, we use accuracy as the final evaluation criterion. The best figure of each metric are highlighted in bold.

Methods	Dataset1			Dataset2		
	Precision	Recall	Accuracy	Precision	Recall	Accuracy
**GoogleNet**	0.8545	**0.9400**	0.9267	0.6090	0.8636	0.8833
**Modified Vgg16**	0.8763	0.8500	0.9100	0.6103	0.7545	0.8771
**Modified ResNet50**	0.8857	0.9300	0.9367	0.6129	0.8636	0.8848
**FireNet**	0.8557	0.8300	0.8967	0.4857	0.8416	0.8309
**Ours**	0.9462	0.8800	0.9433	0.6846	**0.9273**	0.9155
**Ours (Entropy)**	**0.9674**	0.8900	**0.9533**	**0.7239**	0.8818	**0.9232**

## Data Availability

Not applicable.
